# Age Pathognomonic Indicators of Injury Predisposition as a Basis for Public Health Preservation during Physical Activity

**DOI:** 10.3390/ijerph18041989

**Published:** 2021-02-18

**Authors:** Maria V. Sankova, Vladimir N. Nikolenko, Marine V. Oganesyan, Andjela D. Vovkogon, Ekaterina L. Chirkova, Mikhail Y. Sinelnikov

**Affiliations:** 1 Department of Human Anatomy, First Moscow State Medical University Named after I.M.Sechenov (Sechenov University), st. Trubetskaya, 8, bld. 2, 119991 Moscow, Russia; cankov@yandex.ru (M.V.S.); vn.nikolenko@yandex.ru (V.N.N.); marine-oganesyan@mail.ru (M.V.O.); andzelavovk@mail.ru (A.D.V.); ekchirkova82@yandex.ru (E.L.C.); 2 Department of Human Anatomy, Lomonosov Moscow State University, Leninskie Gory, 1, 119991 Moscow, Russia; 3 Research Institute of Human Morphology, Curupa, 3, 117418 Moscow, Russia

**Keywords:** public health preservation, post-exercise injuries, injury predisposition, pathognomonic indicators, age features, injury prevention

## Abstract

A necessary condition for public health maintenance is regular physical activity. A significant increase in the number of musculoskeletal injuries, occurring during physical education and sport activities, actualizes the development of effective measures for their prevention. Early diagnosis of injury predisposition, based on identification of connective tissue dysplasia indicators specific for different age periods, is of particular importance for the prevention of such injuries. The study, performed in accordance to STROBE guidelines, included 78 persons separated into two age subgroups: Group 1 (age 22–35) and Group 2 (age 36–47). Morphometric signs of connective tissue dysplasia and clinical symptoms associated with predisposition to chronic injury were assessed. For persons in Group 1, these indicators included: asthenic body type, joint hypermobility, thin elastic skin, keloid scars, and soft auricles. For the second group: kyphosis, skin hyperpigmentation above the spine, flatfeet, valgus installation, rectus muscles diastasis, atrophic striae, recurrent hernias, and lower-limb varicosity. Universal pathognomonic indicators, such as “crunching” in the temporomandibular joint, gothic palate, altered chest shape, scoliosis, and X- and O-shaped legs are significant at any age. The established pathognomonic indicators will promote early diagnosis of injury predisposition help, and develop effective measures of their prevention and public health preservation during physical activity.

## 1. Introduction

Regular physical activity is necessary for improvement of public health. Regular exercise helps prevent and treat various chronic disorders, including life-threatening cardiovascular and respiratory diseases [1]. A significant increase in the number of musculoskeletal system injuries, occurring during routine physical education and training in the absence of traumatic factors can be seen. Frequent post-exercise injuries lead to the development of chronic post-traumatic conditions of the musculoskeletal system and the loss of physical capacity for an extended period of time [2]. This underlines the importance of the development of effective measures for injury prevention [3]. 

Frequent post-exercise injury is often associated with connective tissue disorders [4]. This pathology is diagnosed by identifying a complex of significant morphometric signs of connective tissue pathology [2]. Dysplastic signs often exacerbate with age, since the time of their manifestation depends on specific patterns of gene expression, and the influence of environmental factors; therefore, there is a certain degree of accumulation defect in the connective tissue system ontogenesis [5]. Early diagnosis of injury predisposition, based on identification of connective tissue dysplasia indicators specific for different age periods, is of particular importance for the prevention of such injuries and improvement of public health. 

Current studies focus on treatment aspects and a diverse abundance of corrective procedures for such injuries, yet a significant gap can be seen in preventative studies and a large cohort of surveillance studies. As such, evaluation of possible diagnostic and preventative treatment targets for injury prevention is of sufficient interest. The goal of this study was to determine age pathognomonic indicators of injury predisposition to develop effective measures for their prevention, including correction of metabolic processes and an individually designed training program. Determining specific factors associated with injury predisposition in different age groups can potentially help form evidence-based recommendations for physical training regimens, preventative therapy, and lifestyle recommendations. More so, individuals with such conditions may have undiagnosed conditions negatively impacting their quality of life. The identifications of such conditions can help improve individual wellbeing and have a substantial impact on public health. 

## 2. Materials and Methods

The current study was approved by the Ethics Committee of First Moscow State Medical University, named after I.M. Sechenov (Sechenov University) under protocol № 08-19 at 5 June 2019. The study was performed in accordance with STROBE guidelines. Seventy-eight participants who were active in amateur (non-professional) running to sustain or improve their health and fitness were included in the study. The inclusion criteria were musculoskeletal system injuries, occurring regularly during routine training, such as joint dislocations and subluxations, ligament sprains or ruptures, bone fractures, bruising, or stiffness. The ages of the surveyed persons was 35.07 ± 5.64 years (range of 22 to 47 years). The participants were split into two age subgroups for a comparative analysis: Group 1 (age 22–35 years), and Group 2 (age 36–47 years).

Clinical-instrumental examination was aimed at identifying morphometric signs of connective tissue pathology according to a questionnaire compiled at the First Moscow State Medical University [4]. Body proportionality was assessed according to Verveka and Pignet indices, body weight was assessed via the Quetelet and Vargi indices, dolichostenomelia was assessed with the arm length/height, upper body/lower body, foot length/height, and wrist length/height ratios. A wrist test, thumb test, and middle finger length were used to diagnose arachnodactyly. Bayton criteria were assessed to confirm the presence of joint hypermobility [6,7]. Instrumental examinations included ultrasound, esophagogastroduodenoscopy, and radiography.

Statistical data analysis was conducted using RStudio (RStudio, Boston, MA, USA). The Kramira-Welch and Fisher RScriteria were used. The differences’ significance level was determined at *p* < 0.05.

## 3. Results

**Figure 1:**
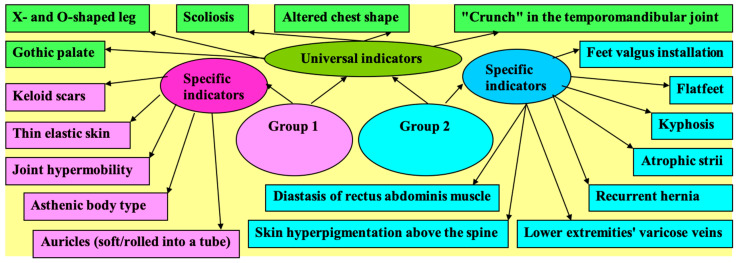
Universal and age-specific pathognomonic indicators of injury predisposition.

The significant findings of our study are summed up in Figure 1. They can be characterized into universal indicators of injury predisposition and age-specific indicators (Figure 1). Age-specific indicators have been shown to be statistically associated with a corresponding age group, prevailing in one group and lacking in incidence in the other.

**Figure 2:**
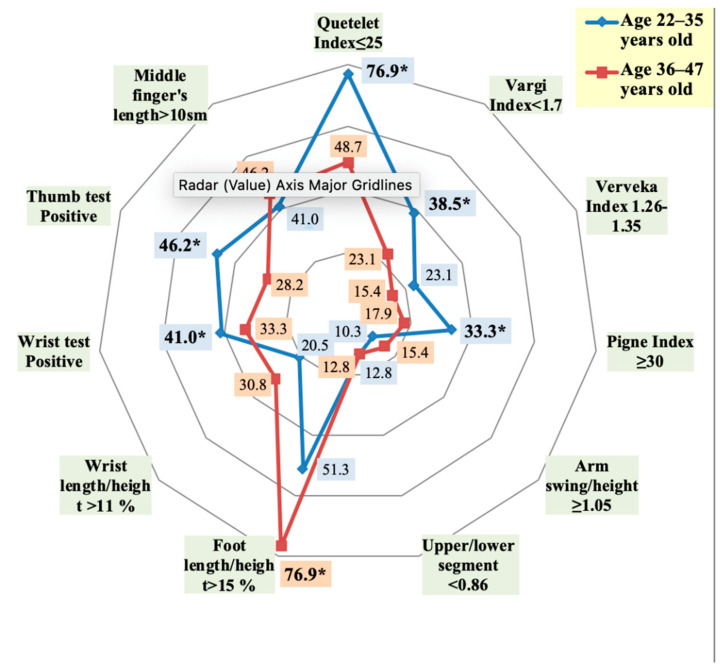
The incidence of the diagnostically significant indices and tests, indicating the presence of asthenic body type, dolichostenomelia, and arachnodactyly in persons with frequent post-exercise injuries of musculoskeletal system depending on age (* statistically significant findings).

The asthenic body-type, identified via the Quetelet, Varga, and Pignet indices, was more common in Group 1, and its diagnostic value significantly decreased with age (Figure 2). Increased bone length (dolichostenomelia) proved to be a leading sign of connective tissue dysplasia. While it was diagnosed in more than half of the subjects (through foot length/height ratio), it was much more prominent in older adults (Group 2).

Frequent sprains and ruptures, as well as spinal pain were seen in all respondents (Figure 2). Joint hypermobility was significantly more prevalent in the younger group (Group 1) (*p* < 0.05). Pathological joint mobility naturally leads to the appearance of non-physiological joint movements and chronic subluxation and dislocation, the incidence of which has shown to have an age-independent distribution. Group 2 has a significantly higher prevalence of joint “crunching”, arthralgia, fractures, flatfoot, valgus deformity, and kyphosis (*p* < 0.05).

**Figure 3:**
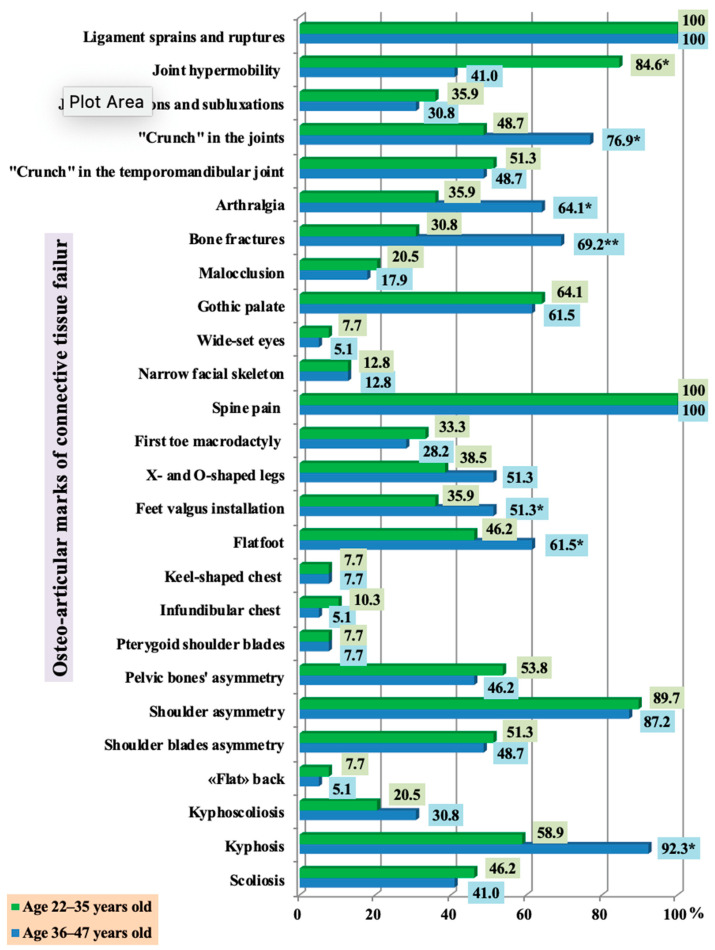
The incidence of osteo-articular marks in persons with frequent post-exercise injuries of the musculoskeletal system, depending on age (* statistically significant findings).

In over half of the subjects with frequent post-exercise injuries, connective tissue failure was evident in such age-independent signs as gothic palate, “crunch” in the temporomandibular joint, scoliosis, changes in the shape of the legs, malocclusion, and chest deformity. Spine and chest pathology, changes in leg shape, and an incorrect motor stereotype can cause functional biomechanical disorders, which are manifested in all respondents by spine pain and may lead to the formation of shoulder ptosis, and asymmetry of shoulder blades and pelvic bones in the majority of study participants, regardless of age (Figure 3). 

**Figure 4:**
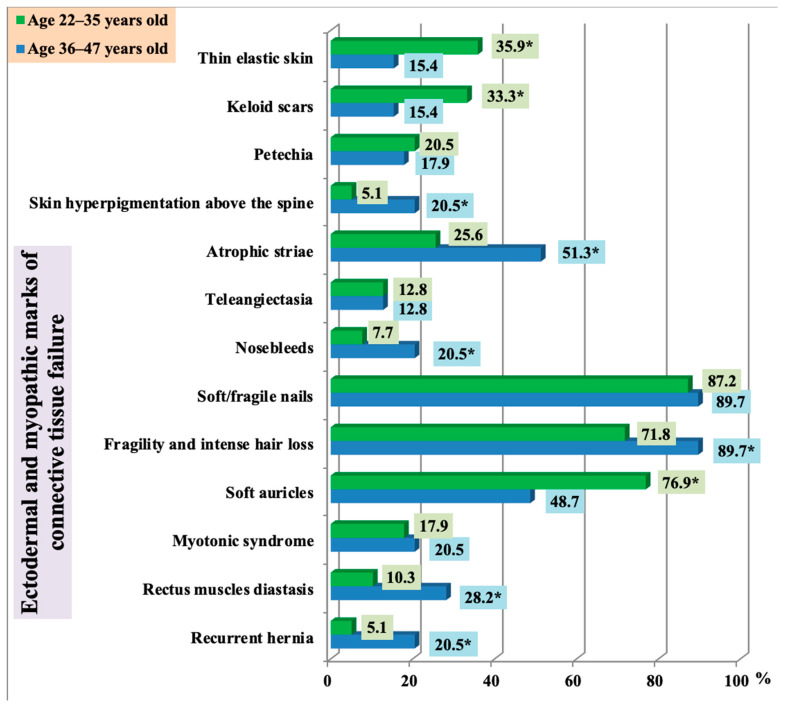
The incidence of ectodermal and myopathic marks in persons with frequent post-exercise injuries of musculoskeletal system depending on age (* statistically significant findings).

Analysis of ectodermal dysplastic stigmas in patients with frequent post-exercise injuries showed that thin elastic skin, keloid scars, and soft curling auricles were more common in younger adults (Group 1). At the same time, atrophic striae, hair fragility, alopecia areas, skin hyperpigmentation above the spine, rectus diastasis, and recurrent hernia were more typical for older adults (Group 2) (Figure 4). These findings were statistically significant (*p* < 0.05).

**Figure 5:**
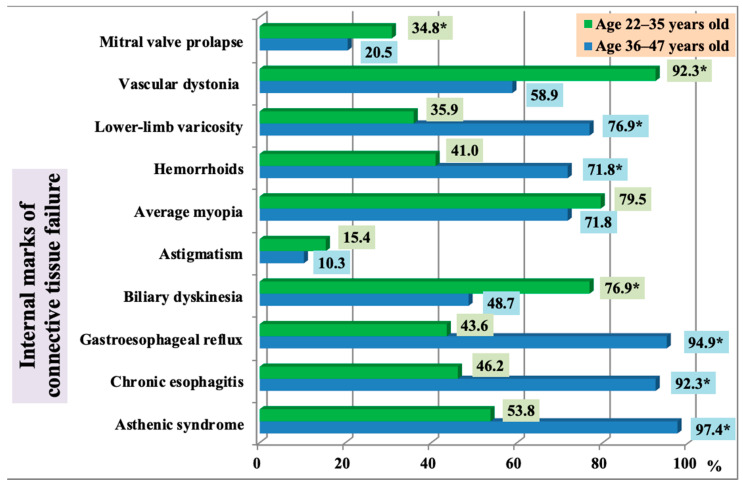
The incidence of internal marks in persons with frequent post-exercise injuries of the musculoskeletal system, depending on age (* statistically significant findings).

Patients in Group 1 more often presented with biliary dyskinesia, mitral valve prolapse, and vascular dystonia (*p* < 0.05). The older adult group (Group 2) prevailed in incidence of lower-limb varicosity, hemorrhoids, gastroesophageal reflux, asthenic syndrome, and chronic esophagitis (*p* < 0.05). Vascular dystonia was very common in younger subjects. The most common complaints were palpitations, headaches, poor tolerance to moderate physical exercise, increased fatigue, and sweating. The older age group (Group 2) had significantly higher blood pressure (systolic 102.2 ± 4.9% and 126.3 ± 7.1% respectively, *р* < 0.05; diastolic 64.3 ± 4.8% and 72.7 ± 5.4% respectively, *р* < 0.05). These findings are presented in Figure 5.

**Figure 6:**
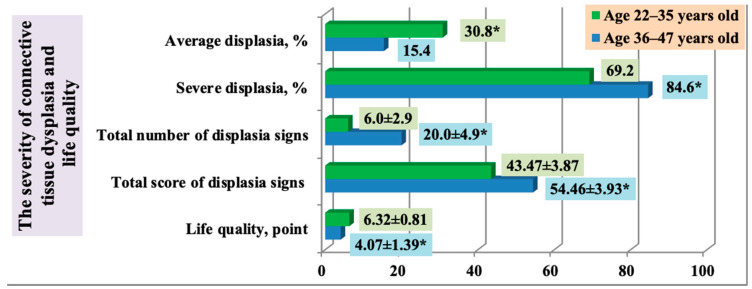
The severity of connective tissue dysplasia and life quality in persons with frequent post-exercise injuries of musculoskeletal system depending on age (* statistically significant findings).

A significant increase in the spectrum and severity of dysplastic changes over ontogenesis determined a higher total score for dysplastic signs and a lower score for life quality in the older age group (Group 2) in comparison with the younger age group (Group 1) (Figure 6).

## 4. Discussion 

Connective dysplasia is characterized by an abundance of manifestations, several of which appear to be age-dependent [8]. Dolichostenomelia is more often diagnosed in adults [9]. This is due to the fact that natural foot-flattening and height decrease occur due to age-related involution [10,11]. Structural disorders of collagen and elastin fibers lead to low strength and elastic properties of the joint capsule and ligaments, which is manifested by frequent sprains and ruptures, as seen in our results. The excessive mobility of the carpometacarpal and metacarpophalangeal joints, characteristic of the younger age group, determines higher positive rates of the wrist and thumb tests, confirming arachnodactyly. With age, hypermobility decreases, due to elastic fiber content decrease and their affinity for calcification. The incongruence of contacting joint surfaces inevitably results in an uneven distribution of biomechanical load. As a result, certain cartilage spaces have to perform inadequate mechanical work, and therefore, the risk of their damage and degeneration increases, which manifests in the early onset of deforming osteoarthritis and “crunch” in the joints. Arthralgia is also more often observed later in life, when dystrophic changes in the tendon-ligamentous apparatus are added to the existing connective tissue defect [5]. 

Chest deformities have been shown to be diagnostically significant signs of dysplastic processes [7,9]. Approximately every fifth person had a degree of chest deformity in our findings. Our results show that progressive structural disorders of the bone and cartilage increase the incidence of kyphotic spinal deviation, flatfeet syndrome, and valgus installation in the older age group, which is confirmed by data of similar studies [5]. Inadequate collagen structure and uneven distribution of mineralization can also create conditions for supporting bone dysfunction in the form of a decrease in density, strength, and elasticity, which may lead to recurrent bone fractures, the prevalence of which was higher in the older age group in our study.

Significant changes in glycosaminoglycan, type I and III collagen of the anterior abdominal wall have been shown to occur with age [11]. This may have determined a greater percentage of rectus muscle diastasis and recurrent hernias in older adults in our study. External signs of connective tissue failure are quite often combined with a specific pathology of internal organs that is connected with the systemic nature of this pathology [12]. Defects in connective tissue components lead to decreased stability and strength of connective tissue structures of various organs, determining clinical symptoms and a variety of complaints [13]. In our study, asthenic syndrome was predominant in the subjects, which may be associated with vegetative imbalance. Every third study participant in Group 1 had mitral valve prolapse. With age, the prevalence of this anomaly decreased significantly. By reducing the elasticity of the valve cusps and the addition of sclerodegenerative changes, this pathology contributes to the development of organic heart disease that was confirmed by other studies [10]. The higher percentage of lower-limb varicosity and hemorrhoids found in our study may be due to progressive structural disorders in the walls of arteries, veins, and their valve apparatus [14].

The cause of dyspeptic complaints, such as heartburn, epigastric pain, heaviness in the liver, and bloating is thought to be autonomic dysfunction, which causes disturbances in the motility of the gastrointestinal tract [15]. It should be noted that persons with frequent post-exercise injuries have a high prevalence of average myopia, which is an example of changes in specific types of connective tissue [16].

The ranking of the most common defects depending on their diagnostic significance made it possible to identify universal and age-specific pathognomonic indicators for early diagnosis of injury predisposition [17]. For persons in Group 2, such indicators became the asthenic body type, with joint hypermobility, thin elastic skin, keloid scars, and soft auricles; for persons of the second adulthood period—kyphosis, skin hyperpigmentation above the spine flatfoot with valgus installation, rectus muscle diastasis, atrophic striae, recurrent hernias, and lower-limb varicosity. Universal pathognomonic indicators, such as “crunch” in the temporomandibular joint, a gothic palate, altered chest shape, scoliosis, and X- and O-shaped legs are important for people of any age.

Limitations of our study include a small number of patients selected from a larger cohort. This can be improved by expanding inclusion criteria and involving several centers in further analysis. A potential confirmation bias was avoided through blinding survey data to exclude patient identification. Further studies should be focused on prevention methods and larger cohort analysis of signs and associations characteristic of recurrent muscle injury. 

**Figure 7:**
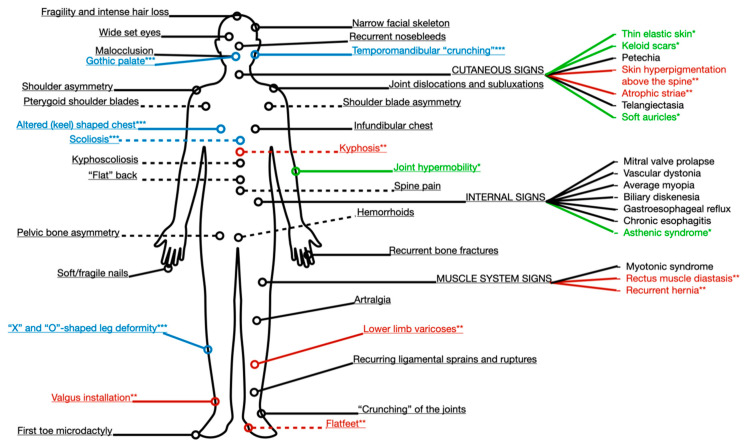
Diagnostic targets to evaluate connective tissue-associated predisposition to recurrent injuries (* green: prevalent and pathognomonic for younger age group, Group 1; ** red: prevalent and pathognomonic for older age group, Group 2; *** blue: significant clinical manifestations for both groups).

The established pathognomonic indicators (Figure 7) necessitate timely changes in the training regime, the correction of metabolic processes, and the appointment of recovery treatment, in particular, osteopathy, which helps strengthen connective tissue and restore the biomechanical functions of the musculoskeletal system [18].

## Data Availability

All associated data is available from the corresponding author upon reasonable request.
